# Simultaneous detection of five shrimp pathogens using a single-tube EvaGreen real-time PCR assay with differential melting temperature

**DOI:** 10.1128/aem.00591-25

**Published:** 2025-08-12

**Authors:** Haoyu Lou, Xuan Li, Guohao Wang, Kaisong Zhang, Kejun Wang, Qiongying Tang, Guoliang Yang, Peng Jia, Jinbo Xiong, Jie Huang, Xuan Dong

**Affiliations:** 1 State Key Laboratory of Mariculture Biobreeding and Sustainable Goods, Yellow Sea Fisheries Research Institute, Chinese Academy of Fishery Sciences, Laboratory for Marine Fisheries Science and Food Production Processes, Qingdao Marine Science and Technology Center, Key Laboratory of Maricultural Organism Disease Control, Ministry of Agriculture and Rural Affairs, Qingdao Key Laboratory of Mariculture Epidemiology and Biosecurity 117919 , Qingdao, China; 2 College of Life Sciences, Huzhou University 117774, Huzhou, China; 3 Jiangsu Shufeng Aquatic Seed Industry Co., Ltd. 117774, Gaoyou, Jiangsu, China; 4 Shenzhen Technology University 507738, Shenzhen, China; 5 State Key Laboratory for Quality and Safety of Agro-Products, School of Marine Sciences, Ningbo University 631318 , Ningbo, China; UMR Processus Infectieux en Milieu Insulaire Tropical, Ste. Clotilde, France

**Keywords:** shrimp pathogens, EvaGreen, multiplex detection, melting temperature

## Abstract

The World Organisation for Animal Health (WOAH) has assessed crustacean diseases, such as infections with white spot syndrome virus (WSSV), infectious hypodermal and hematopoietic necrosis virus (IHHNV), decapod iridescent virus 1 (DIV1), and acute hepatopancreatic necrosis disease (AHPND), as listed diseases, and infection with *Ecytonucleospora hepatopenaei* (EHP) as an emerging disease, all of which significantly threaten the shrimp industry. This study developed a quintuplex EvaGreen-based melting curve real-time PCR method for the simultaneous detection of WSSV, IHHNV, DIV1, AHPND-causing *Vibrio* (*V*_AHPND_), and EHP. In the specific assay, only the target pathogen demonstrated efficient and detectable amplification, thereby indicating that the method exhibits high specificity. Regarding sensitivity testing, the five pathogens were detected at a concentration of 1.0 × 10^1^ copies/μL. Each concentration gradient was evaluated in triplicate, and the coefficient of variation for each gradient remained below 3.38%, thereby affirming that the method demonstrates highly repeatability. We tested the diagnostic sensitivity (DSe) and the diagnostic specificity (DSp) of our method using a total of 800 clinical samples which were gathered from the shrimp farming regions in China. The newly established method in this study demonstrated a DSe above 89.74% for the five pathogens and a DSp of 100%. The quintuplex EvaGreen real-time PCR method developed here offers an accurate and efficient approach for EHP, WSSV, *V*_AHPND_, IHHNV, and DIV1.

**IMPORTANCE:**

Crustacean diseases, such as infections with WSSV, IHHNV, DIV1, *V*_AHPND_, and EHP, pose a significant threat to the global shrimp industry, leading to substantial economic losses. Rapid, accurate, and simultaneous detection of these pathogens is crucial for effective disease management and biosecurity in shrimp farming. In this study, we developed a quintuplex EvaGreen-based melting curve real-time PCR method that enables the simultaneous detection of these five major shrimp pathogens with exceptional specificity, sensitivity, and repeatability. By evaluating 800 clinical samples, our method demonstrated high diagnostic sensitivity and specificity, making it a valuable tool for early pathogen detection and disease control. This novel approach can help mitigate disease outbreaks, improve shrimp farm productivity, and support the sustainable development of the aquaculture industry.

## INTRODUCTION

The aquaculture industry, as a vital part of global food production, has experienced rapid growth in recent years (1, 2). Shrimp play a vital role in aquaculture, encompassing economically valuable species, such as shrimp, crabs, and lobsters (3). However, shrimp farms are facing increasingly severe disease challenges, which have become a critical bottleneck for sustainable development in the industry (4). Various diseases not only cause large-scale mortality events but also severely reduce farming efficiency (5). *Ecytonucleospora hepatopenaei* (EHP), originally named *Enterocytozoon hepatopenaei*, is a microsporidian that primarily targets the hepatopancreas of shrimp (6). While EHP does not directly cause mortality, it substantially hampers the growth rate of shrimp, leading to economic losses for farmers (7, 8). White spot syndrome virus (WSSV) is highly infectious and can spread among various shrimp species, including shrimp and crabs. Once infected, the virus rapidly replicates within the host, forming distinctive white spots on the carapace and ultimately leading to high mortality rates (9, 10). Acute hepatopancreatic necrosis disease-causing *Vibrio* (*V*_AHPND_) mainly infects the gut-associated tissues of shrimp, causing acute necrosis of the hepatopancreatic tissue (11). Infected shrimp typically exhibit clinical signs, such as lethargy, empty intestines and stomach, and extensive shedding of hepatopancreatic epithelial cells (11, 12). Infection with infectious hypodermal and hematopoietic necrosis virus (IHHNV) in shrimp typically results in stunted growth, deformities, and even death, leading to significant reductions in farming yields (13, 14). Infected shrimp may not show distinct and recognizable clinical signs in the early stages, while the pathological changes become more severe as the virus replicates (15). Decapod iridescent virus 1 (DIV1) has significant pathogenicity in shrimp and other crustaceans. Infected shrimp typically exhibit noticeable pathological changes, including whitening of the body, reduced vitality, and high mortality rates (16, 17). The co-infection situation is becoming more and more common, with WSSV and IHHNV co-infection in wild crabs discovered in India in 2021 (18). Hemolymph coagulation in *Penaeus vannamei* is suppressed following infections with WSSV and DIV1 (19). Instances of co-infection with the infectious precocity virus (IPV) and DIV1 have been documented in giant freshwater prawns (*Macrobrachium rosenbergii*) (20).

Effective disease management and control rely heavily on the prompt and accurate identification of pathogens. While traditional diagnostic techniques like histopathology and immunological assays offer certain advantages, they are often limited by lower sensitivity and an inability to detect multiple pathogens at once. In contrast, molecular methods, especially real-time polymerase chain reaction (PCR), have become indispensable for pathogen detection, offering superior sensitivity and specificity. Moreover, the use of real-time PCR in combination with melting temperature analysis, enabled by EvaGreen, provides a more streamlined and cost-effective diagnostic approach (21). These assays enable the concurrent and quantitative identification of multiple targets within a single reaction, thereby enhancing their utility in pathogen diagnostics.

In this study, we established a quintuplex real-time PCR detection method utilizing melting curve analysis with the EvaGreen nucleic acid stain, marking a considerable breakthrough in the detection of shrimp pathogens. This streamlined approach allows the simultaneous identification of a range of pathogens, including EHP, WSSV, *V*_AHPND_, IHHNV, and DIV1, exhibiting excellent performance and reliability. By comparing the results of this novel method with those from the established TaqMan real-time PCR technique, we validated its performance. This method not only offers strong technical support for detection of the targeted pathogens, but also holds significant practical value for early diagnosis and disease control measures.

## MATERIALS AND METHODS

### Experimental samples

The specimens used to evaluate the detection method’s specificity included DNA extracts from EHP, WSSV, *V*_AHPND_, IHHNV, and DIV1, which were used to construct a quintuplex EvaGreen real-time PCR assay. Additional validation of pathogens comprised of IPV, yellow head virus genotype 8 (YHV-8), infectious myonecrosis virus (IMNV*),* Macrobrachium rosenbergii golda virus (MrGV), and covert mortality nodavirus (CMNV), as well as *Vibrio orientalis*, *Photobacterium damselae* subsp. *piscicida*, *V. rotiferianus*, *V. owensii*, and *V. natriegens*, all of which had been isolated from shrimp specimens and archived in our laboratory. These pathogens were pre-verified through TaqMan real-time PCR or standardized diagnostic procedures. To validate the applicability of our quintuplex EvaGreen real-time PCR assay, 800 shrimp specimens were collected across five major aquaculture provinces of Shandong, Jiangsu, Zhejiang, Guangdong, and Hainan in China.

### Primer design

The primers were designed based on the conserved sequences of each pathogen. Specific target genes for design included the gene sequence of the spore wall protein 1 (SWP) from EHP (GenBank ID: KX258197.1) (22), the gene sequence of the WSV313 protein from WSSV (GenBank ID: NC_075105.1) (23), the gene sequence of the PirB protein from *V*_AHPND_ (GenBank ID: KM067908.1) (24), the gene sequence of NS1 protein from IHHNV (GenBank ID: JN616415.1) (25), and the gene sequence of the major capsid protein (MCP) from DIV1 (GenBank ID: KY681039.1) (26). The primer design was conducted using primer-BLAST (https://www.ncbi.nlm.nih.gov/tools/primer-blast/). The primer design process incorporated bioinformatic analysis using MFEprimer3.1 (https://mfeprimer3.igenetech.com/spec) to evaluate potential secondary structures, including intramolecular hairpins and inter-sequence dimers. For each target pathogen, a minimum of 20 primer pairs were computationally designed and subsequently synthesized by Tsingke Biotechnology Co., Ltd., with thermodynamic parameters optimized through the platform’s structural prediction algorithms.

### Construction of the standard plasmids

To construct the standard plasmids for the detection of the five pathogens, the target gene sequences were first amplified by PCR using the specific primers designed for each pathogen. The amplified products were subsequently purified using a gel extraction kit, and the DNA fragments were cloned into the pUC57 vector system, generating recombinant constructs designated as pUC57-*swp*, -*wsv313*, -*pirB*, -*ns1*, and -*mcp*. Following transformation into competent cells, plasmid concentrations were quantified for subsequent applications. Plasmid DNA was extracted from the selected colonies using a mini-prep kit and confirmed by PCR and sequencing to ensure the correct insertion of the target gene sequences. The verified plasmids were then quantified using a spectrophotometer and stored at −20°C for future use.

### Optimization of the quintuplex EvaGreen real-time PCR assay

To determine the best experimental conditions, we varied primer concentrations (from 0.08 μM to 0.6 µM) and annealing temperatures (ranging from 55°C to 65°C). The optimal conditions were selected based on the comparison of C_t_ values, the melting curve peaks, and the interactions between primers. Each condition was performed in triplicate for consistency.

The real-time PCR reaction mixture consisted of 12.5 µL of TOROGreen HRM qPCR Master Mix (TOROIVD, China), along with specific primer concentrations for each pathogen. For WSSV and IHHNV, 1 µL of each forward and reverse primer (10 µM) was used. For *V*_AHPND_ and DIV1, 0.8 µL of each forward and reverse primer (10 µM) was added, and for EHP, 1.4 µL of each forward and reverse primer (10 µM) was included. The reaction also contained 1 µL of template nucleic acid, with nuclease-free water added to bring the total volume to 25 µL.

The thermal cycling conditions included an initial denaturation step at 95°C for three minutes, followed by 40 cycles of two-step amplification: denaturation at 95°C for 10 seconds and annealing at varying temperatures (55–66°C, eight variants) for 20 seconds. Melting curve analysis was performed by gradually increasing the temperature: 95°C for 15 seconds, followed by 64°C for 60 seconds, and then continuous fluorescence monitoring from 68°C to 85°C with 0.5°C increments.

### Construction of standard curves

The standard plasmids of pUC57-*swp*, -*wsv313*, -*pirB*, -*ns1*, and -*mcp* were subjected to 10-fold serial dilutions. Serial decimal dilutions spanning 10^1^ to 10^8^ copies/μL (10-fold dynamic range) served as templates. The standard curve was generated by plotting logarithmic plasmid concentrations versus corresponding C_t_ values.

### Sensitivity testing

The extracted recombinant plasmids pUC57-*swp*, -*wsv313*, -*pirB*, -*ns1*, and -*mcp* were diluted to the same concentration, achieving a final plasmid concentration of 100 ng/µL. Then, 10-fold serial dilutions were prepared using ddH_2_O, with concentrations of 1 × 10^1^−1 × 10^8^ copies/µL. These served as DNA templates for sensitivity testing of the EvaGreen-based quantitative PCR method to assess its detection sensitivity. The detection limit of the template copy number was determined by the instrument’s ability to detect the minimum fluorescence signal.

### Specificity testing

The simultaneous detection specificity of the EvaGreen-based real-time PCR assay was thoroughly validated by conducting an ASp assessment using nucleic acids from EHP, DIV1, WSSV, IHHNV, *V*_AHPND_, IMNV, CMNV, *V. orientalis*, *P. damselae* subsp. *piscicida*, *V. rotiferianus*, *V. owensii*, *V. natriegens*, YHV-8, IPV, and MrGV, along with ddH_2_O and negative control using nucleic acids from healthy *M. rosenbergii*. The extracted viral DNA was tested using the aforementioned EvaGreen real-time fluorescent PCR detection. Viral RNA was extracted and reverse transcribed into cDNA, which was subsequently analyzed using the established EvaGreen real-time PCR detection method in this work. ddH_2_O was included as a blank control, while DNA extracted from positive samples of EHP, WSSV, *V*_AHPND_, IHHNV, or DIV1 served as positive control.

### Repeatability testing

To assess both intra-assay and inter-assay repeatability, different concentration gradients of pUC57-*swp*, -*wsv313*, -*pirB*, -*ns1*, and -*mcp* plasmids (spanning from 1 × 10^1^−1 × 10^8^ copies/µL) were utilized in the experiments. Each concentration was repeated three times for accuracy. Intra-assay repeatability was evaluated by performing three repetitions of each concentration, and the experiment was tested in triplicate. Repeatability was quantified using the coefficient of variation (CV), which is calculated as the percentage ratio of the standard deviation to the mean C_t_ value for each concentration.

The optimized multiplex real-time PCR parameters (quintuplex format) were executed on a Bio-RAD CFX96 thermocycler, with post-amplification repeatability assessed through quantitative analysis of cycle threshold metrics using Microsoft Excel.

### Co-infection testing

To prove that the method can detect multiple pathogens at the same time, different combinations of nucleic acids from two or more pathogens were prepared and tested for their positive rates using the quintuplex EvaGreen real-time PCR. ddH_2_O was included as the blank control, and each group was evaluated in triplicate.

### Testing of clinical samples

The TaqMan real-time PCR detection methods for EHP (27), WSSV (28), *V*_AHPND_ (29), IHHNV (30), and DIV1 (31) were used to compare the positive rates, diagnostic sensitivity (DSe), and specificity (DSp) with our quintuplex EvaGreen Real-time PCR assay. The DSe and DSp were calculated according to the guidelines outlined in WOAH (2024).

## RESULTS

### Primer optimization

An initial set of 100 primer pairs was evaluated, and the first selection criterion was the ability of the primers to clearly distinguish between the five pathogens based on their distinct melting temperatures. The next step was to verify that no primer dimers formed between any of the five pairs. During the selection process, we prioritized primers that demonstrated high amplification efficiency, focusing on those that resulted in reduced C_t_ values and minimal variation in standard deviations (SD) during real-time PCR assays. The final panel of optimized primers for the simultaneous detection of all five pathogens using quintuplex EvaGreen real-time PCR is presented in Table 1.

**TABLE 1:** Primers designed in this study for the identification of EHP, WSSV, *V*_AHPND_, IHHNV, and DIV1

Primer	Sequence (5′−3′)	Amplicon size (bp)	Tm (°C)
EHP-2-F	CGAGTTTGGCGGCACAATTCTCA	109	73.8–74.0
EHP-2-R	TCCTACAAATGCTGTGTCTGTGTAA		
WSSV-Y-2F	GGGAGATCTTCGAACCCTGG	213	74.8–75.0
WSSV-Y-2R	TGAATCTCGGCACACACTCG		
VP-PB-3F	CGCGAGCTAGACGGTGATGAATGGC	82	76.7–76.9
VP-PB-3R	ATCAGCCCACGCAGCGAGTTCT		
IHHNV-P-3F	CCAGGCAAGGTGGGACTCCG	182	78.6–79.0
IHHNV-P-3R	TCGCGCTCTAAGTGACGGCG		
DIV1-M-4F	ACCGTGGCTCTCCCAGTCGGTGGT	176	80.0–80.1
DIV1-M-4R	GCGTGTGAGGGGGCAACGGCGATA		

### Establishing optimal reaction parameters

**Fig 1:**
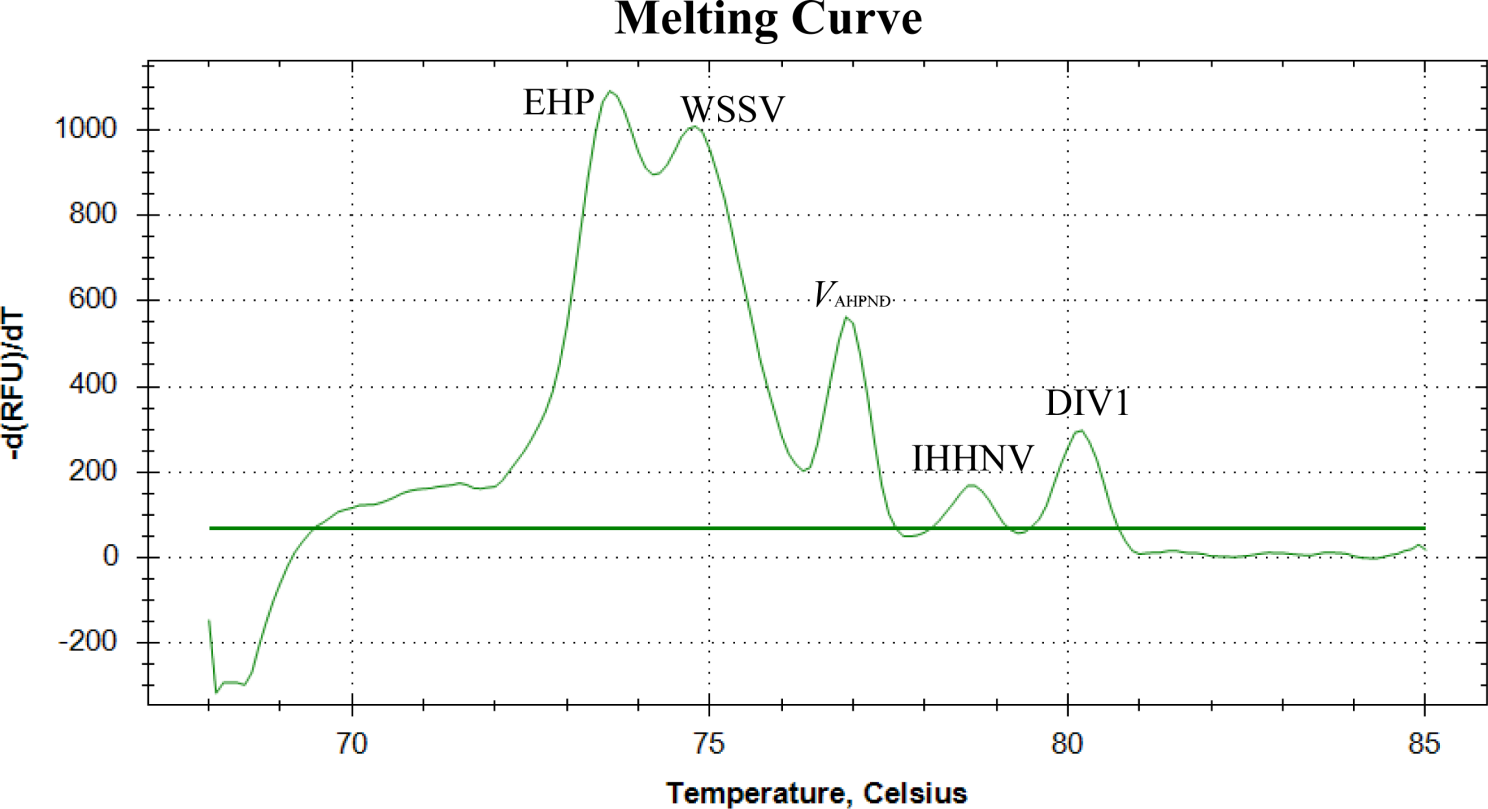
Melting curves of the quintuplex EvaGreen-based melting curve real-time PCR method. EHP, *Ecytonucleospora hepatopenaei*; WSSV, white spot syndrome virus; *V*_AHPND_, acute hepatopancreatic necrosis disease-causing *Vibrio*; IHHNV, infectious hypodermal and hematopoietic necrosis virus; DIV1, decapod iridescent virus 1.

The C_t_ values of primer concentrations are shown in Table 2. Then, primer concentrations were carefully optimized based on the comparison of C_t_ values, the characteristics of melting curve peaks, and the interactions between primers. The final optimized concentrations for the five pairs of primers for EHP were 0.52 µM, the concentration for WSSV and IHHNV primers was 0.4 µM, and the concentration for *V*_AHPND_ and DIV1 primers was 0.32 µM. The optimal annealing temperature for the reactions was determined to be 63.3°C (Table 3). We generated five melting curves of the quintuplex EvaGreen-based real-time PCR method using a mixed DNA template containing all five target pathogens (Fig. 1).

**TABLE 2:** C_t_ values of primer concentration optimization

Pathogen	C_t_ value (mean ± standard deviation) at primer concentration of:					
	0.08 µM	0.2 µM	0.32 µM	0.4 µM	0.52 µM	0.6 µM
EHP	30.07 ± 0.06	28.39 ± 0.07	27.67 ± 0.00	27.27 ± 0.03	27.04 ± 0.00	27.34 ± 0.30
WSSV	ND^*a*^	32.33 ± 0.22	27.37 ± 0.00	26.60 ± 0.03	25.42 ± 0.54	25.21 ± 2.10
*V*_AHPND_	27.33 ± 0.28	24.41 ± 1.08	23.03 ± 0.00	22.87 ± 0.11	22.12 ± 0.04	22.18 ± 0.03
IHHNV	34.12 ± 0.95	30.88 ± 0.83	29.58 ± 0.08	29.47 ± 0.04	29.64 ± 0.11	28.99 ± 0.00
DIV1	27.99 ± 0.18	25.31 ± 0.12	23.20 ± 0.07	23.24 ± 0.20	22.58 ± 0.02	22.35 ± 0.03

^*a*^ ND, not detectable.

**TABLE 3:** C_t_ values of annealing temperature optimization

Pathogen	C_t_ value (mean ± standard deviation) at annealing temperature of:							
	65°C	64.5°C	63.3°C	61.4°C	59°C	57°C	55.7°C	55°C
EHP	27.33 ± 0.68	27.49 ± 0.64	27.60 ± 0.62	28.08 ± 0.24	27.76 ± 0.10	28.00 ± 0.15	27.98 ± 0.27	28.07 ± 0.06
WSSV	17.16 ± 0.04	17.28 ± 0.06	17.15 ± 0.01	17.58 ± 0.03	17.83 ± 0.01	18.50 ± 0.21	18.48 ± 0.25	18.64 ± 0.15
*V*_AHPND_	27.78 ± 0.06	27.16 ± 0.00	26.81 ± 0.00	28.42 ± 4.95	27.08 ± 0.01	27.45 ± 0.05	27.72 ± 0.13	28.93 ± 2.25
IHHNV	22.01 ± 0.00	21.80 ± 0.01	20.79 ± 0.01	20.72 ± 0.59	20.01 ± 0.00	27.54 ± 0.01	19.65 ± 0.00	20.87 ± 2.05
DIV1	22.08 ± 0.09	22.41 ± 0.05	22.13 ± 0.03	22.53 ± 0.02	22.49 ± 0.01	22.71 ± 0.02	22.99 ± 0.12	22.89 ± 0.04

### Establishing standard curves and sensitivity testing

**Fig 2:**
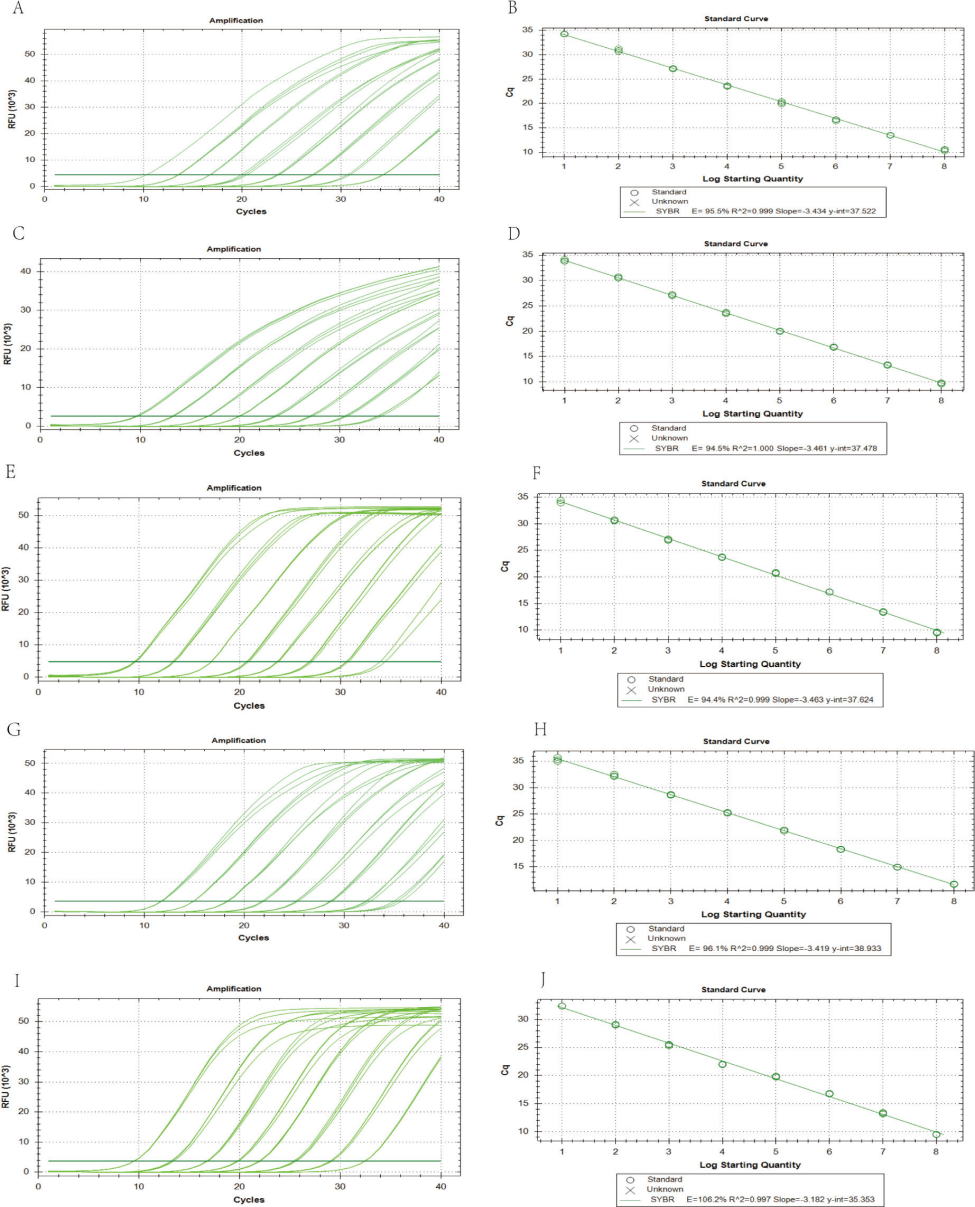
Amplification curve and standard curve of quintuplex EvaGreen real-time PCR. (A, C, E, G, and I) Amplification curves of the recombinant plasmids of the five pathogens (EHP, WSSV, IHHNV, *V*_AHPND_, and DIV1). (B, D, F, H, and J) Standard curves of the recombinant plasmids of the five pathogens (1 × 10^1^ to 1 × 10^8^ copies/µL).

Employing the optimized quintuplex EvaGreen real-time PCR assay, plasmid standards for five pathogens (EHP, WSSV, *V*_AHPND_, IHHNV, DIV1) underwent decade-fold serial dilution (10^1^–10^8^ copies/μL) to establish standard curves. Amplification data demonstrated linear responses across this dynamic range (*R*^2^ ≥0.997), with log-linear correlations between template concentrations and C_t_ values (Fig. 2). All targets exhibited optimal amplification efficiencies (94.4–106.2%) within the validated quantification range. The following were derived: for EHP (y = −3.434x + 37.522, *R*^2^ = 0.999, *E* = 95.5%), for WSSV (y = −3.461x + 37.487, *R*^2^ = 1, *E* = 94.5%), for *V*_AHPND_ (y = −3.463x + 37.624, *R*^2^ = 0.999, *E* = 94.4%), for IHHNV (y = −3.419x + 38.933, *R*^2^ = 0.999, *E* = 96.1%), and for DIV1 (y = −3.182x + 35.353, *R*^2^ = 0.997, *E* = 106.2%). The assay demonstrated sensitivity down to 10^1^ copies per reaction for each of the pathogens EHP, WSSV, *V*_AHPND_, IHHNV, and DIV1, with the respective C_t_ values recorded as 34.71, 33.79, 34.19, 35.47, and 35.08, respectively.

### Specificity testing

**Fig 3:**
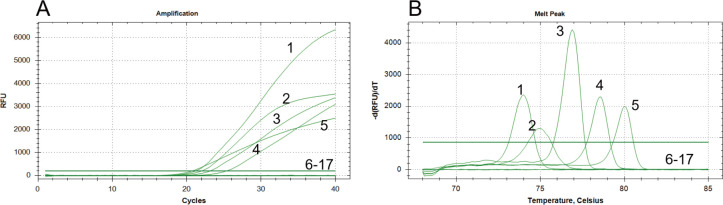
Specificity analysis of quintuplex EvaGreen quintuplex real-time PCR in this study. (**A**) Amplification curves. (**B**) Melting curves. 1–5: EHP; WSSV; *V*_AHPND_; IHHNV; and DIV1. 6–17: *Vibrio orientalis*, *Photobacterium damselae* subsp. *piscicida*, *V. rotiferianus, V. owensii, V. natriegens*, IPV, IMNV, YHV-8, CMNV, MrGV, blank control, and negative control

By employing the quintuplex EvaGreen real-time PCR method outlined, amplification of positive DNA templates for the five pathogens resulted in clear and specific amplification curves. However, no amplification was detected for *V. orientalis*, *P. damselae* subsp. *piscicida*, *V. rotiferianus*, *V. owensii*, and *V. natriegens*, as well as the cDNA from IPV, IMNV, YHV-8, CMNV, MrGV, blank control, and negative nucleic acids. The quintuplex EvaGreen real-time PCR assay demonstrated high specificity, exhibiting no specific amplification of other pathogens during validation (Fig. 3).

### Repeatability testing

To evaluate the repeatability of the method, plasmid standards for five pathogens (EHP, WSSV, *V*_AHPND_, IHHNV, DIV1) were subjected to decade-fold serial dilution (ranging from 10^1^ to 10^8^ copies/μL) as amplified samples. The evaluation results showed that within the starting amount of template varying from 1.0 × 10^1^ to 1.0 × 10^8^ copies/μL, the inter-assay and intra-assay coefficients of variation for EHP plasmids (pUC57-*swp)* were less than 0.02% and 2.95%. The inter-assay and intra-assay coefficients of variation were less than 0.78% and 3.38% for WSSV plasmids (pUC57-*wsv313)*; less than 0.83% and 3.31% for *V*_AHPND_ plasmids (pUC57-*pirB)*; less than 1.07% and 3.11% for IHHNV plasmids (pUC57-*ns1)*; and less than 0.97% and 1.00% for DIV1 plasmids (pUC57-*mcp)*, respectively (Table 4).

**TABLE 4:** Variability in intra-assay and inter-assay results of the quintuplex EvaGreen real-time PCR assay

Plasmid	Concentration (copies/μL)	Intra-assay C_t_			Inter-assay C_t_		
		x̅	SD	CV (%)	x̅	SD	CV (%)
pUC57-*swp*	1.0 × 10^8^	10.17	0.30	2.95	10.47	0.18	0.02
	1.0 × 10^7^	13.15	0.29	2.23	13.48	0.03	0.00
	1.0 × 10^6^	16.31	0.29	1.75	16.60	0.16	0.01
	1.0 × 10^5^	19.85	0.29	1.44	20.15	0.28	0.01
	1.0 × 10^4^	23.31	0.28	1.19	23.57	0.09	0.00
	1.0 × 10^3^	26.76	0.35	1.29	27.14	0.08	0.00
	1.0 × 10^2^	30.48	0.37	1.21	30.90	0.32	0.01
	1.0 × 10^1^	34.22	0.06	0.16	34.22	0.03	0.00
pUC57-*wsv313*	1.0 × 10^8^	10.37	0.10	1.01	10.32	0.08	0.78
	1.0 × 10^7^	13.93	0.14	0.98	13.88	0.05	0.36
	1.0 × 10^6^	17.15	0.18	1.05	17.03	0.01	0.06
	1.0 × 10^5^	20.24	0.29	1.45	19.99	0.04	0.20
	1.0 × 10^4^	23.60	0.53	2.24	23.14	0.08	0.35
	1.0 × 10^3^	26.91	0.80	2.96	26.10	0.08	0.30
	1.0 × 10^2^	30.67	1.04	3.38	29.50	0.13	0.44
	1.0 × 10^1^	33.79	1.08	3.20	32.55	0.03	0.09
pUC57-*pirB*	1.0 × 10^8^	9.94	0.33	3.31	10.00	0.08	0.80
	1.0 × 10^7^	13.63	0.21	1.54	13.64	0.14	1.03
	1.0 × 10^6^	17.18	0.06	0.36	17.13	0.08	0.47
	1.0 × 10^5^	20.53	0.18	0.86	20.55	0.17	0.83
	1.0 × 10^4^	23.72	0.03	0.13	23.73	0.04	0.17
	1.0 × 10^3^	26.99	0.10	0.37	27.07	0.09	0.33
	1.0 × 10^2^	30.47	0.19	0.61	30.28	0.08	0.26
	1.0 × 10^1^	34.19	0.40	1.16	34.15	0.03	0.09
pUC57-*ns1*	1.0 × 10^8^	11.80	0.37	3.11	11.67	0.06	0.51
	1.0 × 10^7^	15.03	0.42	2.77	14.92	0.03	0.20
	1.0 × 10^6^	18.47	0.49	2.67	18.30	0.06	0.33
	1.0 × 10^5^	21.90	0.65	2.95	21.90	0.12	0.55
	1.0 × 10^4^	25.22	0.62	2.44	25.23	0.10	0.40
	1.0 × 10^3^	28.58	0.65	2.29	28.65	0.08	0.28
	1.0 × 10^2^	32.25	0.66	2.06	32.34	0.24	0.74
	1.0 × 10^1^	35.47	0.65	1.84	35.35	0.38	1.07
pUC57-*mcp*	1.0 × 10^8^	10.59	0.24	2.25	10.77	0.02	0.19
	1.0 × 10^7^	14.32	0.23	1.58	14.43	0.14	0.97
	1.0 × 10^6^	17.84	0.23	1.27	17.91	0.07	0.39
	1.0 × 10^5^	21.20	0.47	2.21	20.94	0.09	0.43
	1.0 × 10^4^	24.09	0.83	3.43	23.14	0.03	0.13
	1.0 × 10^3^	27.50	0.83	3.02	26.66	0.15	0.56
	1.0 × 10^2^	31.08	0.84	2.69	30.23	0.09	0.30
	1.0 × 10^1^	35.08	1.00	2.85	35.12	0.02	0.06

### Co-infection testing results

**Fig 4:**
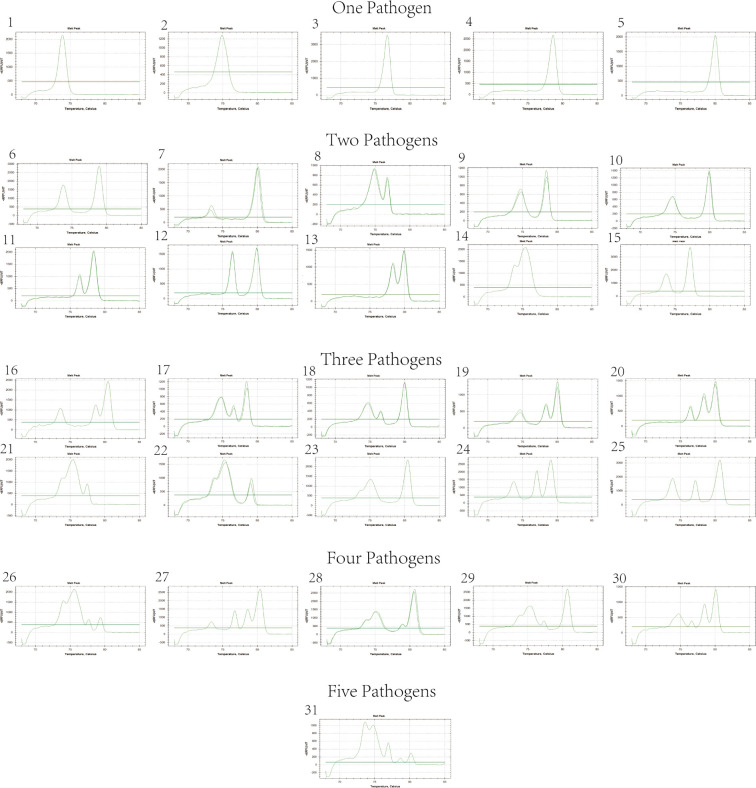
Melting curves of different combination patterns of the five pathogens.

The quintuplex EvaGreen real-time PCR assay was used to detect different combinations of two or more viral nucleic acids, providing a comprehensive assessment of co-infection scenarios. The assay successfully identified all possible combinations, demonstrating its versatility and reliability in diagnosing multiple infections simultaneously. The results were all positive, confirming the presence of the targeted pathogens, and the dissolution curve peaked at the corresponding position (Fig. 4), indicating the specificity and sensitivity of the assay. This capability of detecting multiple pathogens in a single test is particularly valuable for understanding their potential synergistic effects on disease severity.

### Testing of clinical samples

Samples from 800 shrimp samples were subjected to testing. The positive rate of the quintuplex EvaGreen real-time PCR detection for EHP was 12.5% (100/800), WSSV was 5.25% (42/800), *V*_AHPND_ was 5.50% (44/800), IHHNV was 4.38% (35/800), and DIV1 was 11.25% (91/800). The co-infection rate of WSSV and DIV1 detected by the quintuplex EvaGreen real-time PCR was 3% (24/800), while that of EHP and IHHNV was 0.88% (7/800). The findings revealed that the C_t_ values for the positive samples in the quintuplex EvaGreen real-time PCR assay varied from 15.24 to 38.68. This quintuplex EvaGreen real-time PCR technique effectively detected more than 96% of the positive samples identified by the singleplex TaqMan real-time PCR, as shown in Table 5. In contrast to the detection methods advocated by WOAH, the method newly developed in this research has achieved a DSe of 98.0% and a DSp of 100% for the detection of EHP. For the detection of WSSV, the method has DSe of 91.3% and DSp of 100%. For the detection of *V*_AHPND_, the method has DSe of 95.7% and DSp of 100%. For the detection of IHHNV, the method has DSe of 89.7% and DSp of 100%. For the detection of DIV1, the method has DSe of 98.9% and DSp of 100% (Table 6).

**TABLE 5:** Detection results in clinical samples^*a*^

Pathogen	Positive detection rate		Accuracy rate
	Quintuplex EvaGreenReal-time PCR	TaqMan Real-time PCR	
EHP	12.50% (100/800)	12.75% (102/800)	98.04%
WSSV	5.25% (42/800)	5.75% (46/800)	91.30%
*V*_AHPND_	5.50% (44/800)	5.75% (46/800)	95.65%
IHHNV	4.38% (35/800)	4.88% (39/800)	89.74%
DIV1	11.25% (91/800)	11.50% (92/800)	98.91%
WSSV and DIV1 co-infection	3.00% (24/800)	3.00% (24/800)	100%
EHP and IHHNV co-infection	0.88% (7/800)	0.88% (7/800)	100%

^*a*^ The total number of samples was 800.

**TABLE 6:** Diagnostic sensitivity and diagnostic specificity of the quintuplex EvaGreen real-time PCR

Pathogen	No. of reference samples	
	Diagnostic sensitivity	Diagnostic specificity
EHP	100/102 × 100% = 98.04%	38/38 × 100% = 100%
WSSV	42/46 × 100% = 91.30%	94/94 × 100% = 100%
*V*_AHPND_	44/46 × 100% = 95.65%	94/94 × 100% = 100%
IHHNV	35/39 × 100% = 89.74%	101/101 × 100% = 100%
DIV1	91/92 × 100% = 98.91%	48/48 × 100% = 100%

## DISCUSSION

In recent years, as shrimp farming has become more intensive, the occurrence of co-infections by multiple pathogens has been on the rise (32–34). Therefore, this study designed five pairs of specific primers based on the conserved gene sequences of these five pathogens, which successfully establishes a quintuplex EvaGreen real-time PCR assay. This method is not only more convenient but also significantly more cost-effective than probe-based methods, with each sample costing less than 1 Chinese Yuan—a substantial reduction in cost compared to traditional methods. The entire reaction process takes only 1 hour and 24 minutes, saving time compared to the conventional molecular method, which requires separate testing for each pathogen. This streamlined and economical approach provides valuable assistance for the detection of these five pathogens, making it a preferred alternative for laboratories and clinics with budget constraints or limited resources.

The TaqMan real-time PCR for EHP, IHHNV, DIV1, WSSV, and *V*_AHPND_ can all detect up to 10^1^ copies/μL and even 10^0^ copies/μL (35–39). In addition, a multiplex PCR has been developed for simultaneously detecting WSSV and hepatopancreatic parvovirus (HPV), with a sensitivity reaching up to 10^2^ copies/μL (40). The quintuplex EvaGreen real-time PCR established in this study had a sensitivity that was comparable with the TaqMan real-time PCR. However, the former approach is cheaper and faster for detecting the five pathogens, which reduces the risk of contamination associated with opening the lid compared to conventional PCR. Additionally, the type of pathogen infection can be determined by the value of the melting curve. Of particular significance is the scalability of our method, which allows for the incorporation of additional pathogens beyond the initial panel. In contrast, probe-based assays face practical limitations when detecting five or more pathogens due to constraints in probe availability and escalating costs, potentially impeding their applicability to extend coverage to a broader range of pathogens.

Multiplex real-time PCR technology plays an increasingly important role in clinical diagnosis due to its high sensitivity and specificity. The specificity of the method was evaluated by testing DNA from common shrimp pathogens, such as *V. orientalis*, *P. damselae* subsp. *piscicida*, *V. rotiferianus*, *V. owensii*, and *V. natriegens*, as well as cDNA from IPV, IMNV, YHV-8, CMNV, and MrGV, along with blank control and negative controls. The results demonstrated a high level of specificity. The method also exhibited good repeatability for the five pathogens, with CV values below 3.38%.

The elevated detection rates of the five pathogens in our clinical samples across different provinces can be linked to the deliberate selection of samples exhibiting disease symptoms. The identification of co-infections, such as the simultaneous presence of WSSV and DIV1 within individual samples, underscores the intricate nature of disease interactions within shrimp populations, emphasizing the need for thorough diagnostic strategies. Analysis of samples with different concentrations and mixed infections revealed that the melting curves of the five pathogens have different intervals. The melting curve intervals for EHP, WSSV, *V*_AHPND_, IHHNV, and DIV1 are 73.8–74.0°C, 74.8–75.0°C, 76.7–76.9°C, 78.6–79.0°C and 80.0–80.1°C, respectively. If the machine model is changed, the peak value of the melting curve may be biased, which needs to be further determined by positive samples. This indicates that the method can identify and differentiate the five pathogens through melting curves. Moreover, during infections with two or more pathogens, the melting curves can appear corresponding to the curves, greatly reducing the time required for the detection of multiple pathogens.

### Conclusions

This study established a Tm value-based quintuplex EvaGreen real-time PCR detection method, which has high sensitivity, specificity, and repeatability, enabling the simultaneous detection of EHP, WSSV, *V*_AHPND_, IHHNV, and DIV1. Notably, the method is also applicable for the detection of mixed pathogens, making it highly suitable for laboratory diagnostics and epidemiological surveillance.
